# Minimally invasive Knapp's procedure: Modified fornix incision approach

**DOI:** 10.4103/0301-4738.57148

**Published:** 2009

**Authors:** Jitendra Jethani

**Affiliations:** Pediatric Ophthalmology and Strabismus Clinic, Dr. Thakorbhai V Patel Eye Institute, Vadodara, India.

Dear Editor,

Minimally invasive surgical procedures reduce tissue trauma, postoperative patient discomfort, hospital stay, working disability, and the economic impact of surgery.[1,2] The majority of surgeons use the limbal approach incision in squint surgery, first described by Harms[3] in 1949 and later popularized by von Noorden.[4] A lot of incisions have been described for simple recession and resection of muscles. Small and micro incisions have been advocated in strabismus surgery too.[5–6] Traditional incision for Knapp's procedure is a large limbal U-shaped incision[5] similar to the limbal incision but slightly larger.[1,2] We describe Knapp's procedure via fornix incisions to make it a minimally invasive conjunctival approach. The incisions may be left unsutured too since they would be in the cul de sac.

**Figure 1:**
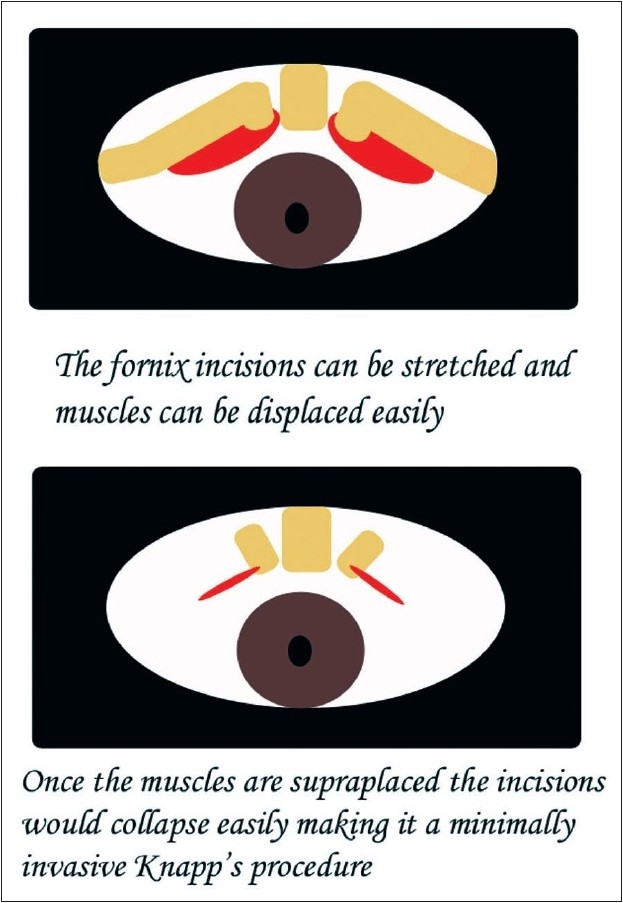
Schematic diagram shows the incision site and the displacement of the muscles (here yellow). The two oval red marks represent the incision (in the lower picture) after the surgery is complete

**Figure 2:**
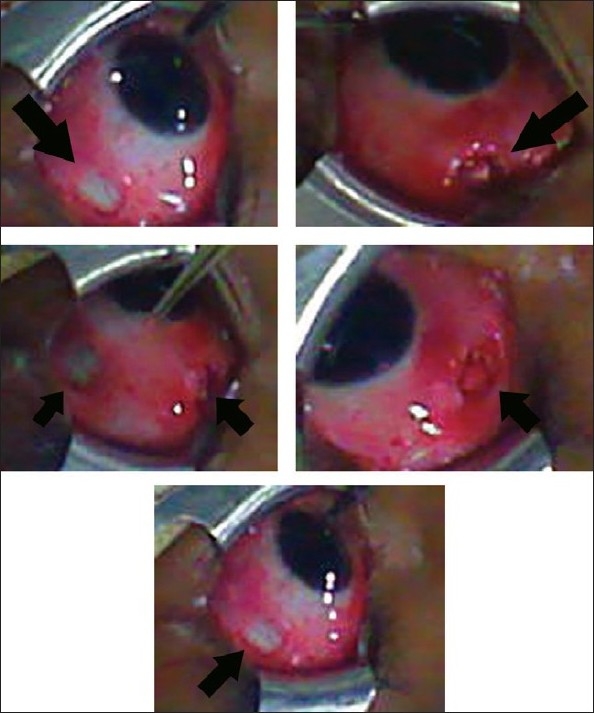
Various photographs showing the small buttonhole-like incision created in the upper nasal and upper temporal fornices. The arrows show the incision site

**Figure 3:**
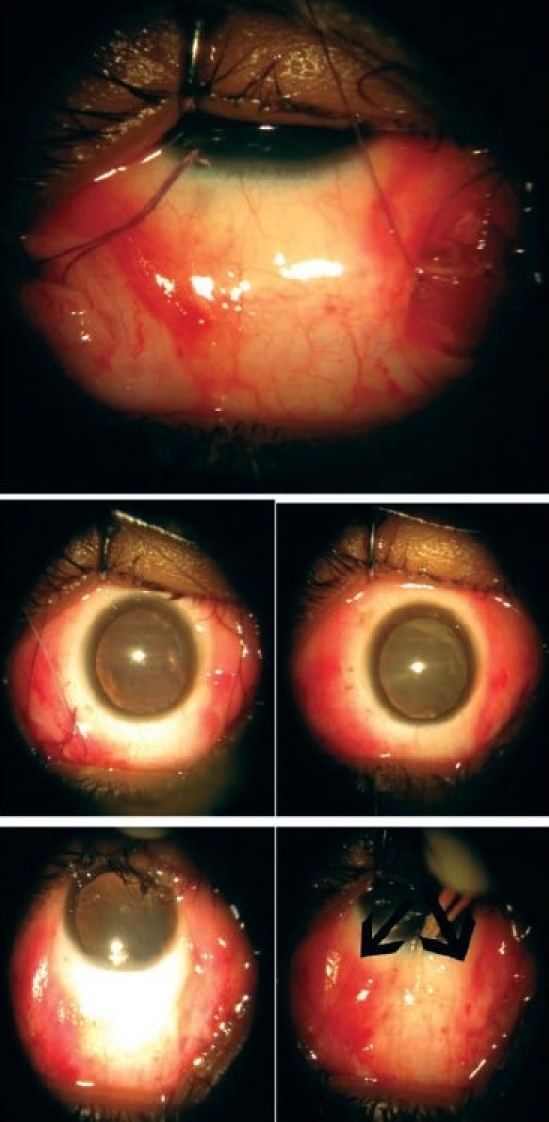
Another patient who underwent Knapp's procedure via fornix route. The superior photograph shows the muscle reattached parallel to the superior rectus. The lower right photograph shows the final outcome after the incisions have been sutured. The arrows show the incision sites

**Figure 4:**
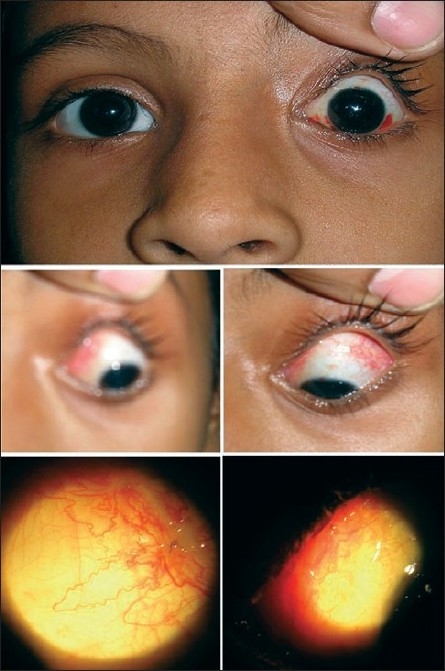
Postoperative pictures one week after Knapp's procedure. There is minimal congestion surrounding the incision site well hidden by the lid

The patient was prepared for the surgery under general anesthesia. After putting the speculum in place, a 6-0 vicryl stay suture was passed at 10.30 o' clock in right eye and the eyeball was pulled at an angle. Around 8 mm from the limbus in the superotemporal quadrant a conjunctival incision concentric to the limbus was placed [Fig. 1]. The conjunctiva and Tenon's was separated and the lateral rectus muscle was hooked. The muscle was freed from its attachments with the conjunctiva, intermuscular ligament and 6-0 vicryl sutures were passed at the insertion. The muscle was cut at the insertion and resutured back near the superior rectus concentric to the limbus. A similar incision was put in the superonasal quadrant and the medial rectus was hooked, sutured, separated and reinserted parallel to the superior rectus on the nasal side concentric to the limbus [Figs. 1–3]. At the end of the surgery only two incisions in the upper fornix were seen [Figs. 1, 2] which can be sutured with a single 8-0 vicryl suture or may be left unsutured. The postoperative period showed small, localized areas of redness in the upper nasal and temporal quadrants [Fig. 4].

A large range of incisions, limbal, cul de sac and more recently minimally invasive incisions have been described in strabismus surgery.[5–6] Minimally invasive strabismus surgery (MISS) is becoming popular. The purpose of these smaller and smaller incisions is to reduce the hyperemia, for better healing and earlier patient rehabilitation.[6]

In a study of comparison of limbal incision with the minimally invasive incision, Mojon *et al*.,[6] found that the MISS technique was better than the limbal approach as the former had less dissection, less chances of limbal ischemia and therefore less postoperative tissue scar. However, with a fornix incision all the merits of MISS are available as pointed out by Kushner.[7] We, therefore used the fornix incision for the classic Knapp's procedure and called it minimally invasive Knapp's procedure. Such a possibility has also been hinted at by Coats *et al*., who have told about both the larger incision and small buttonhole incisions for transposition surgeries.[8] Such incisions, by leaving the limbal conjunctiva undisturbed, may play a role in reducing the risk of anterior segment ischemia.[9] Since a MISS incision could be possibly used for such large displacement of muscles, fornix incision would be a great alternative to the large limbal incision.

The incision for Knapp's procedure is usually a large U-shaped incision starting from the inferior temporal quadrant to the inferonasal quadrant at the limbus (from 8 o'clock to 4 o'clock) (that is around 270°). Such a large incision would cause a lot of dissection and a lot of scar tissue formation postoperatively as a result of the healing process. Also, the conjunctiva superiorly may lead to peripheral opacification of superior cornea.[5]

The cul de sac incision helps in minimizing the bleeding, reduces the unnecessary dissection and may help in earlier healing and therefore earlier rehabilitation of the patient postoperatively. One of the limitations is that the amount of exposure may be slightly reduced. However, we feel that a trained surgeon using a cul de sac incision routinely would find it very easy to put two such separate incisions. Such openings may not allow performing full rectus muscle transposition in older patients with inelastic conjunctiva.

Two small cul de sac incisions can be used to perform a Knapp's procedure. This approach may help in earlier patient rehabilitation and reduced scar formation in patients undergoing Knapp's procedure; however, this needs further research and clinical trials.
